# 

**Published:** 1892-05

**Authors:** 


CONTENTS

VOL. 39. No. 5.

| | PAGE. |
|---|---|
| A Word With Our Readers | 97 |
| Talks with Dr. Mandeville | 97 |
| At the Moment of Death | 100 |
| Rescued by his Horse | 101 |
| How is it Done ? | 103 |
| Sunstroke—How to Treat it | 104 |
| Alcohol and Insanity | 105 |
| Whole Wheat Bread | 106 |
| School Girls' Luncheons | 107 |
| Meat Eaters | 108 |
| One or Two in a Bed | 109 |
| Helpful Ailments | 110 |
| The Sabbath | 111 |
| How Women Bathe in Paris | 112 |
| Bromide of Potash and the Liver | 113 |
| Shrewd Trick of a Dog | 114 |
| The Tobacco Habit | 114 |
| Swedish Cure for Drunkenness | 115 |
| Miscellaneous | 115 |
| Literary | 118 |

INDEX TO ADVERTISEMENTS OF HALF A PAGE AND OVER.

| | PAGE. |
|---|---|
| Herba Vita | I |
| Hall's Journal | II |
| Bovinine | IV |
| Ayer's Pills | V |
| Hall's Journal Premiums | VII |
| Washington Improvement Co. | VIII |
| Hall's Premium List | X |
| Royal Baking Powder | XI |
| Buffalo Lithia Water | XI |

